# Effects of Fennel Seed Powder Supplementation on Growth Performance, Carcass Characteristics, Meat Quality, and Economic Efficiency of Broilers under Thermoneutral and Chronic Heat Stress Conditions

**DOI:** 10.3390/ani10020206

**Published:** 2020-01-26

**Authors:** Ahmed A. AL-Sagan, Shady Khalil, Elsayed O. S. Hussein, Youssef A. Attia

**Affiliations:** 1 King Abdulaziz City for Science & Technology, P.O. Box 6086, Riyadh 11442, Saudi Arabia; abdeen@kacst.edu.sa; 2 AlzChem Trostberg GmbH, 83308 Trostberg, Germany; shady.khalil@alzchem.com; 3 Department of Animal Production, College of Food and Agriculture Sciences, King Saud University, Riyadh 11451, Saudi Arabia; shessin@ksu.edu.sa; 4 Arid Land Agriculture Department, Faculty of Meteorology, Environment, and Arid Land Agriculture, King Abdulaziz University, Jeddah 21589, Saudi Arabia

**Keywords:** broilers, heat stress, fennel seed, performance, carcass characteristics, meat quality

## Abstract

**Simple Summary:**

Nowadays, great attention has been given to phytogenic products as a growth enhancer due to their safety and eco-friendly influences on animal nutrition. Thus, this investigation looked at the use of fennel seed powder at 0, 1.6, and 3.2% as a dietary additive on the performance, carcass traits, meat quality, and efficiency of the production of broiler chickens raised under thermoneutral and chronic heat stress conditions. In essence, 3.2% fennel seed powder in the diet of broilers enhanced the growth rate under chronic heat stress and decreased breast meat redness and temperature, suggesting that 3.2% fennel seed could be used as an agent for enhancing the broiler’s tolerance during chronic heat stress (CHS) condition from 19 to 41 days of age.

**Abstract:**

Nowadays, phytogenic products have received great attention as a growth promoter due to their safety and environmentally friendly effect as a replacement for classical growth promoters such as antibiotics in animal nutrition. Thus, this research seeks the possibility of using fennel seed powder (FSP) as a dietary additive from 19 to 41 days of age on productive performance, carcass traits, meat quality, and production efficiency of broiler chickens raised under thermoneutral and chronic heat stress conditions. Thus, 216 one-day-old Ross-308 broiler chicks were divided into two equal groups. The first group was placed in an independent temperature-controlled room at 23 ± 2 °C. The broiler chicks from the second group were placed in a heat-stressed room and exposed to chronic heat stress conditions (32 ± 2 °C) for seven hours per day from 8 a.m. to 3 p.m. The experimental design was 2 × 3 factorial including two environmental temperatures (thermoneutral vs chronic heat stress) and three experimental diets that contained 0, 1.6, and 3.2% FSP. The chickens were randomly assigned to 18-floor pens per room temperature, representing six replicates per treatment and six birds per replicate. The results showed that dietary fennel seed powder during days 19–41 of age enhanced the growth rate of broiler chickens and improved breast meat redness and reduced temperature under chronic heat stress. In conclusion, 3.2% of fennel seed powder could be used as an agent for enhancing the broiler’s tolerance during chronic heat stress condition from 19 to 41 days of age. Moreover, it is necessary to study in further detail the nitrite and nitrate contents in FSP and their impacts on muscle redness (a*) as well as muscle temperature.

## 1. Introduction

In hot regions, the poultry industry is significantly affected by high ambient temperatures, especially when combined with high relative air moisture contents (humidity) [1,2,3]. A further increase of 0.6–2.5 °C is expected to occur due to global warming over the next 50 years [4]. Optimum temperatures for broiler performance are 34–32, 32–28, 28–26, 26–24, 18–24, and 18–24 °C for the first, second, third, fourth, fifth, and sixth weeks of age, respectively [5]. Therefore, it is necessary to reevaluate poultry management to minimize heat stress.

Signs of heat stress begin to appear when the environmental temperature increases above 30 °C [6]. High temperatures can cause serious physiological disturbances [7]. Since birds do not have sweat glands, they evaporate their excess sweat through panting [8]. It has been estimated that approximately 0.5 kcals of energy are lost per gram of water evaporated during panting [9]. Urinary output is another mechanism that accompanies the excreta, with an increase in water intake to reduce body heat. This deviation in normal physiology reduces broiler productivity and leads to economic losses [9]. Generally, heat stress is considered as an inducer of oxidative stress [3,10,11] and can negatively affect animal performance and meat quality [12,13]. Moreover, nutritional intervention, along with proper management, is required to relieve the negative effect of heat stress [1]. Nutritional management can include feed restriction and dietary antioxidant supplementation [14,15] as well as the maintenance of electrolyte balance [16].

Recently, there has been great interest in phytochemicals, which are effective stress-alleviating agents [17,18]. The use of phytochemicals from medicinal plants in poultry diets is more related to product quality and consumer health than to production efficiency [19]. Fennel (Foeniculum vulgare, Apiaceae) seeds contain health-promoting volatile essential oil compounds such as fenchone, anethole, myrcene, limonene, chavicol, cineole, anisic aldehyde, and pinene as well as amino acids, phenolic compounds, and flavonoids [20]. These seeds also have apoptotic, hepatoprotective, antithrombotic, antiviral, antimicrobial, antispasmodic, anti-inflammatory, antimutagenic, antipyretic, and antinociceptive properties [14,20]. These active ingredients are also known to have digestive, anti-flatulent, and carminative properties [21]. This medicinal plant is a potential source of natural phytochemicals, especially antioxidants [22]. Furthermore, it can be used as a therapeutic agent to alleviate heat stress and stress-related diseases [15]. Fennel seeds contain high levels of nitrites and nitrates (65 to 376.7 mg/100 g), which are known to play crucial roles in maintaining vascular and digestive functions [23]. In mammals, nitrates are reduced to nitrites and eventually to nitric oxide (NO), which plays an essential role in the maintenance of the cardiovascular system [23]. In mammals and poultry, NO plays an important role in the regulation of various physiological functions that are mediated by the hypothalamus such as thermoregulation, fever, water balance, and cardiovascular regulation [24]. It has been found that inducible nitric oxide synthase (iNOS) increases in cold stress, heat stress, and inflammatory response [25] and as a response to lipopolysaccharide injection [26]. Since NO is produced from arginine metabolism [27,28], it can be speculated that fennel seed powder (FSP) may supply some NO, helping to reduce arginine metabolism for NO production [29].

Therefore, the objective of this study was to evaluate the potential of different levels of fennel seed powder supplemented to broiler diet as a physiological stimulator for the growth and development of chicks through its impacts on production performance traits, carcass characteristics, digestive organs, and meat quality under thermoneutral and heat stress conditions.

## 2. Materials and Methods

### 2.1. Animals and Treatments

King Abdulaziz City for Science and Technology, Riyadh, Saudi Arabia approved the experimental procedures. It recommends animal rights, welfare, and minimal stress and did not cause any harm or suffering to animals according to the Royal Decree M59 in 14/9/1431H.

Two hundred and sixteen one-day-old Ross-308 broiler chicks were purchased from the Al-Wadi company (Saudi Arabia) and used in this experiment. The chicks were housed in thermostatically controlled rooms. They were fed with a commercial starter mash based on corn and soybean ration (crude protein (CP), 23.2%; metabolizable energy (ME), 12.97 MJ/kg) during day 1 to 18 of age, and were managed according to the Ross-308 broiler guide for husbandry and health care practice.

On day 19, the broilers were divided into two equal groups. The first group was placed in an independent temperature-controlled room at 23 ± 2 °C. The broiler chicks from the second group were placed in a heat-stressed room and exposed to chronic heat stress conditions (32 ± 2 °C) for seven hours per day from 8:00 a.m. to 3:00 p.m. The indoor relative humidity and temperature under thermoneutral conditions during the experimental period for the broiler was 51.2% and 23.1 °C, 57% and 23.9 °C, and 53% and 24.7 °C during days 19–26, 27–33, and 34–41 of age, respectively. Under chronic heat stress conditions, the indoor relative humidity and temperature was 44.9% and 31.5 °C, 47.8% and 31.8 °C, and 41.8% and 31.9 °C during days 19–26, 27–33, and 34–41 of age, respectively. The experimental diets were formulated according to [30], as shown in Table 1. The nitrate contents were calculated using the minimum average reported by [23].

The experimental design was 2 × 3 factorial including two environmental temperatures (thermoneutral vs. chronic heat stress), and three experimental diets that contained 0, 1.6, and 3.2% FSP. The two rooms were equipped with ultrasonic humidifiers to supply constant relative humidity to each room. The chickens were randomly assigned to 18-floor pens per room temperature, representing six replicates per treatment. Each floor pen (0.75 × 0.75 m) had six birds. Fennel seeds were purchased from the local market (imported from India) and ground into a fine powder using an electric mill. The FSP was added to the experimental diet in a mash form at the top. To assure that the diets were consumed, we offered a per weighted amount of feed daily according to the expected feed intake of each pen. Before, next meal, we collect the residual and mixed well with the new amount. From our observation, birds consumed almost all feeds and excessed 97% daily. The provision of feed and water was ad libitum throughout the experimental period. The chicks were exposed to continuous light from 1–6 days of age, then to 23:1 light-dark cycle throughout the experimental period. Feeding on experimental diets was initiated at 19 days of age.

### 2.2. Measurements

#### 2.2.1. Production Performance Measurements

Live body weight (LBW), feed intake (FI) as fed basis, and feed conversion ratio (FCR) were measured for each floor pen every week. Then, the data were averaged for each pen to calculate the FI, body weight gain (BWG), and the FCR for the entire experimental period (19–41 days of age), where the BWG and FCR were calculated as follows:

BWG = Final body weight at 41 days of age-initial body weight at 19 days of age.

FCR = Amount of feed consumed (g)/live weight of chicks (g).

Nitrate intake was calculated using the feed intake data and the average concentration (220.85 mg/100 g) of nitrate in fennel seeds [23] and the available literature data.

Dietary electrolyte balance = Na concentration × 10000/ molecular weights of Na+K concentration × 10000/molecular weights of K- chloride × 10000/ molecular weights Cl.

Nitrate intake = Amount of feed consumed × concentration of nitrates in feedstuffs.

European production index and economic efficiency were determined, according to [18].

#### 2.2.2. Carcass and Meat Characteristics

On day 41, six chicks were randomly taken from each treatment, one per replicate to represent all treatment replicates, individually weighed, and slaughtered. The chicks were scalded at 54 °C for 2 min and then de-feathered, and finally, their heads were removed. After de-feathering, breast muscles and abdominal fat were immediately removed from the hot carcass and weighed. The internal organs were removed in a precise anatomical manner from the beginning of the esophagus to the end of the outlet [31].

The intestines were cleaned and weighed using a 0.1 g sensitive balance, and the data were expressed as a percentage of carcass weight. The clean carcass weight (dressing yield) was expressed as a percentage of live body weight. The dressing percentage was determined by dividing the clean carcass weight by the live body weight. Abdominal fat, leg weight, breast weight, intestinal weight, gizzard weight, liver weight, and heart weight were determined as a percentage of carcass weight, as indicated by [32]. 

#### 2.2.3. pH, Temperature, and Meat Color

The pH of the boneless breast fillets was measured at 15 min post-mortem, using an electronic TPX-90i pH meter (Toko Chemical Laboratories Co. Ltd., Tokyo, Japan) with a needle-type electrode (CE201S-SR; Toko Chemical Laboratories Co. Ltd., Tokyo, Japan). Each sample was measured at three points, and their mean value was recorded. The breast muscle temperature was determined 15 min after slaughter, with a portable digital thermocouple (EcoScan Series, Temp JKT, Eutech Instruments, Vernon Hills, IL, USA). Then, the CIE color parameters (L* (lightness), a* (redness), and b* (yellowness) values) were determined at three points of the breast muscle sample surface on the dorsal side using a CR-400 colorimeter (Konica Minolta Sensing Inc., Osaka, Japan).

#### 2.2.4. Economic Efficiency

Economic efficiency was calculated according to [33]. The assessment was carried out using the income-cost divided by the cost and multiplied by 100. The income is equal to the selling price of body weight at the end of the experiment. The cost included the cost of day-old chicks, veterinary care and other husbandry costs, and cost of feeding. The difference between the income and cost is the net revenue, which is divided by total cost and multiplied by 100 to obtain the economic efficiency.

### 2.3. Statistical Analysis

The general linear model procedure of the SAS Software (Cary, NC, USA) [34] was used for the analysis of the obtained data. A 3 × 2 factorial experiment with six replicates was used in a completely randomized block design. The replicate was the experimental unit. Percentage data were transformed to arcsine before the analysis. Differences in treatment means were compared using the Bonferroni Test at *p* < 0.05.

## 3. Results

### 3.1. Growth Performance

Table 2 shows the effects of the inclusion of FSP in Ross-308 broiler diets and heat stress on BWG, FI, FCR, survival rate, and production index. The interaction effect (Temp × FSP) was observed on the BWG and production index. Under normal temperatures, no differences were noticed among the groups. However, under heat stress, the highest BWG and production index were recorded in the 3.2% FSP (*p* < 0.05). These results reflect the positive effects of feeding the chicks with a diet containing 3.2% FSP on the BWG and production index under heat stress conditions.

Heat stress significantly decreased feed intake and impaired FCR compared to the thermoneutral group. However, the effect of FSP and the interaction between fennel and heat stress were not significant.

### 3.2. Carcass Characteristics

Table 3 presents the carcass characteristics affected by the inclusion of FSP in broiler diets under heat stress conditions. The results indicate that the percentages of dressing, abdominal fat, leg, gizzard, and liver were not significantly different among the heat stress groups and/or the dietary FSP groups. However, significant differences were observed in the percentages of breast muscle due to FSP diets, and in the intestine of broiler chicks due to the interaction effects (Table 3). The highest percentage of the intestine was observed in broilers fed with diets tested under thermoneutral conditions. Groups on CHS had significantly decreased heart percentage compared to the thermoneutral groups. The heart was significantly smaller in broilers fed the 1.6% fennel diet compared to the unsupplemented control. The breast was significantly greater in broilers fed the 1.6% fennel diet compared to the unsupplemented control. 

### 3.3. Meat Quality

The results of nitrate intake, pH, and L*, a*, and b* values as well as temperature are shown in Table 4. Nitrate intake was significantly affected by temperature and levels of FSP. Nitrate intake increased with increasing FSP within each temperature, and significantly decreased due to exposure to high temperature. CHS had a significant impact on broiler meat quality. Breast muscle pH was neither affected by CHS nor by dietary supplementation of FSP (*p* > 0.05). Breast muscle color of broilers subjected to heat stress, as indicated by lightness (L* values), redness (a* values), and yellowness (b* values), had variable responses compared to those under normal temperature conditions. An interaction effect was observed in the redness (a*) of breast muscles (*p* < 0.05). A significant difference between dietary treatments were found under normal temperature conditions (1.6 vs. 3.2 FSP), but non-significant differences under heat stress. 

The dietary supplementation of FSP under both environmental conditions did not affect the yellowness (b* values) and lightness (L* values) of breast meat, but the effect of temperature on the b* and L* values was significant. The value of b* significantly decreased with CHS, while the L* value increased (Table 4).

Breast muscle temperatures were significantly influenced by heat stress and/or dietary FSP supplementation. The highest value (29.67 °C) was observed in broilers fed with the control diet under CHS. Moreover, broilers supplemented with 1.6 and 3.2% FSP had significantly lowered breast temperatures than the unsupplemented control.

### 3.4. Economic Efficiency

Table 5 shows the impact of temperature, funnel supplementation, and the interaction between them on economic efficiency. As expected, CHS had significant negative effects on the economic efficiency traits of broilers. On the other hand, funnel fortification had significant impacts on the total revenue, net revenue, and economic efficiency, showing a positive effect of 3.2% FSP. The interaction effect was significant for most of the traits, except for economic efficiency. The results indicate that feeding 3.2% FSP under CHS increased the feeding cost and total cost compared to other FSP levels. Total and net revenue was significantly decreased due to feeding 1.6% FSP under thermoneutral compared to other FSP levels. In addition, feeding 3.2% FSP under CHS increased the total and net revenue compared to 1.6% FSP.

## 4. Discussion

The main effect of the temperature on broiler performance was significant and confirmed the success of heat stress induction. The adaptation to heat stress markedly influences broiler performance, production index, and economic efficiency [3,13,35]. Broilers at market weight generate approximately 5–10 kcals of energy per hour as a normal physiological process [9]. Under heat stress, broilers dissipate heat from their body to the surrounding environment, assuming that it is at a lower temperature than the body (41 °C) [9]. As body temperature rises above 41.5 °C, broilers are considered to be under heat stress, and their performance is more likely to be adversely affected. However, mortality usually occurs when the temperature is above 42 °C [9].

To lose the extra heat gained from the environment, birds must reduce the FI to decrease metabolic heat production from feed, and thus, their BWG decreases [16]. FSP intake promotes BWG and FCR. Likewise, dietary FSP supplementation significantly increased the growth of quail chicks [16]. In the present study, the best BWG, FCR, breast muscle, and liver percentage were obtained when broilers under normal condition were supplemented with 3.2% of FSP. It is worth noting that FI significantly decreased under CHS conditions among all treatments when compared to the control. The improved performance of broilers on FSP diets may be due to enhanced digestibility and enriched antioxidants status. Fennel also has strong antiviral, antimicrobial, and anti-inflammatory effects, which may improve gut health and eliminate pathogens. It was reported that the FSP is a rich source of essential oil (anethole, fenchone, methyl chavicol, limonene, phellandrene, camphene, pinine, anisic acid, and palmitic, oleic, linoleic, and petroselenic acids, volatile compounds, flavonoids, phenolics [19,20,21].

Our results showed significant alterations in broiler performance, production, and economic efficiency under CHS. This finding is in line with those reported by [13,16]. Dietary supplementation with 3.2% FSP improved broiler growth rate by 4.7% under CHS, while, under optimum temperatures, FSP-supplemented groups displayed similar growth, feed intake, FCR, production index, and economic efficiency. Moreover, our findings were in agreement with those of [20,36]. However, diets with fennel powder of 1, 2, and 3 g/kg resulted in significant improvements in broiler growth and FCR, while feed intake was unaffected [28]. Heat stress may cause a physiological dysfunction that triggers the body to utilize nutrients to synthesize critical proteins instead of using them for growth, which allows broilers to decrease against the oxidative damage of heat stress [2,3,37]. 

The results obtained on the carcass characteristics in the experimental conditions did not show differences in these characteristics, except for heart percentage, which was affected by heat stress. Supplementation of 1.6% and 3.2% FSP seemed to improve breast percentage. Treatments with FSP, particularly at 1.6%, tended to decrease heart percentages. The results of carcass characteristics in this study were in line with those obtained by [38], who reported that the addition of FSP at 1, 2, and 3 g/kg to the diet resulted in insignificant differences for all carcass characteristics. Similar results were also reported by [20]. The results indicate that FSP had no adverse effects on carcass traits. Moreover, phytogenic plants and their essential oils are used as preservation approaches to enhance the sensory attributes and prolong the shelf life of animal products [19,21,36,39].

The results showed that meat quality parameters such as meat color, which is an important visual criterion, were significantly affected by heat stress. The color of meat is correlated with myoglobin and hemoglobin concentrations and their status [40,41]. The current results are in agreement with the results reported by [3,42]. The highest value of a* was reported in the 1.6% FSP group under normal temperatures. However, it was only similar to the control group under the same conditions. It is worth noting that the a* value is extremely important to consumers.

Furthermore, a high redness (a*) value yields an undercooked appearance. The a* value can be affected by bird age, stress before slaughter, and consumption of dietary nitrates [43]. Nitrates have been reported to be at high concentrations (65 to 376.7 mg/100 g) in FSP [23,44,45,46], which may explain the obtained results and have both beneficial and adverse effects [47]. The change in a* value parallels the change in nitrate intake, which increases with increasing FSP concentration and decreased under CHS condition. The nitrate intakes ranged from 56.2 in the control groups to 276 mg/ bird during 19–41 days of age, with an average daily intake of nitrate ranging from 2.44 to 12 mg/day. The toxic level of nitrate for chickens ranges from 450–900 ppm and is 658 ppm for nitrite [30]. Thus, the intake of nitrate was far from the toxic level. The concentration of nitrate in animal feedstuffs is considered safe at 4, 44, and 22 mg/kg in soybean meal, oat grain, and maize grain, respectively [48,49]. Thus, nitrite poisoning is rare in poultry fed cereal/grain/oilseeds meal based diets [50,51,52]. 

Additionally, NO was known to react with a myoglobin increased a* value [27]. Nevertheless, the latter may act as pro- or anti-oxidant, according to its concentration. With higher NO concentration, it may rapidly react with superoxide radical forming proxy nitrite [47], which oxidizes myoglobin, resulting in low a* value [43]. On the other hand, low a* values may indicate more oxidized myoglobin in birds subjected to heat stress, as reported by [53]. 

Heat stress increases breast meat temperatures, which may accelerate oxidative rancidity and increase the peroxidation biomarker. Similarly, CHS was found to increase body temperature in a study conducted by [54]. However, in our study, the FSP-supplemented groups showed significant reductions in breast muscle temperature under both normal and heat stress conditions, revealing a beneficial effect of FSP. This finding, along with the increase in BWG in broilers under CHS and fed 3.2% FSP diet, can be explained on the basis that FSP contains NO, which increases blood flow to the body surface and upper respiratory tract to dissipate body temperature, as reported by [53], suggesting that FSP may be used as a CHS alleviating agent.

In the present investigation, no differences were observed in the pH of meat among different temperatures and/or fennel groups. The pH is one of the most important physical traits for the qualitative profile of meat and is commonly utilized as an assessment of sensory qualities of the technological properties of meat. Meat pH is related to the water-holding capacity [40,55], which is correlated positively with the texture, juiciness, and flavor of meat [56]. 

## 5. Conclusions

According to the findings of this study, the dietary inclusion of FSP, especially at 3.2%, resulted in a beneficial impact on the growth performance and carcass quality of broilers under heat stress conditions. Furthermore, CHS significantly decreased the production index and economic efficiency, but FSP did not affect both criteria.

## Figures and Tables

**Table 1:** Ingredients and nutrient composition of finishers (days 19 to 41), diets as fed basis.

Ingredients and Composition, g/kg	Fennel, g/kg		
	0	16	32
Corn	621	611	602
Soybean meal, 48% CP	287	283	277
Gluten Meal	30.0	30.0	30.0
Fennel seed powder	0.0	16.0	32.0
Palm oil	24.4	22.8	21.8
Dicalcium phosphate	17.5	17.1	16.8
Limestone	7.0	6.8	6.8
Dl-methionine	0.6	0.7	0.8
Lysine-HCl	1.9	2.0	2.2
L-threonine	0.5	0.6	0.7
Premix ^1^	5.0	5.0	5.0
Salt	4.6	4.5	4.4
Choline chloride, 60%	0.5	0.5	0.5
Calculated composition			
ME, MJ/kg ^2^	13.18	13.18	13.18
Dry Matter, g/kg ^3^	894	894	894
Crude protein, g/kg ^2^	205	204	204
Digestible methionine, g/kg ^2^	5.18	5.23	5.28
Digestible lysine, g/kg ^2^	11.0	11.0	11.0
Digestible methionine + cysteine, g/kg ^2^	8.4	8.4	8.4
Digestible threonine, g/kg	7.2	7.2	7.2
Digestible arginine, g/kg	11.6	11.4	11.2
Nitrate, mg/kg	19.5	54.6	89.7
Nitrite, mg/kg	NA	NA	NA
Ether extract, g/kg ^3^	46.2	44.3	42.9
Crude fiber, g/kg ^3^	51.6	52.4	53.8
Calcium, g/kg ^2^	8.5	8.5	8.5
Available Phosphorus, g/kg ^2^	4.2	4.2	4.2
Magnesium, g/kg	1.59	1.59	1.60
Potassium, g/kg	7.93	8.24	8.54
Na (Sodium), g/kg	2.0	2.0	2.0
Cl (Chloride), g/kg	3.78	3.73	3.69
Dietary electrolyte balance, mEq/kg	183	193	201

^1^ Vitamin-mineral premix contains the following per kg: vitamin A, 2,400,000 IU; vitamin D, 1,000,000 IU; vitamin E, 16,000 IU; vitamin K, 800 mg; vitamin B1, 600 mg; vitamin B2, 1600 mg; vitamin B6, 1000 mg; vitamin B12, 6 mg; niacin, 8000 mg; folic acid, 400 mg; pantothenic acid, 3000 mg; biotin 40 mg; antioxidant, 3000 mg; cobalt, 80 mg; copper, 2000 mg; iodine, 400; iron, 1200 mg; manganese, 18,000 mg; selenium, 60 mg, and zinc, 14,000 mg; DL-methionine, 32 g; lysine, 25 g. NA, data not available in addition.

**Table 2:** Broiler body weight gain, feed intake, and feed conversion ratio following supplementation with fennel seed powder under thermoneutral or heat stress conditions.

Temp	Diet	BWG (g/bird)	FI (g)	FCR (g Feed/g Weight Gain)	Survival Rate (%)	European Production Index
Normal	Control	2149 ^a^	3226	1.50	100	341 ^a^
	FSP (1.6%)	2092 ^a^	3137	1.50	100	332 ^a^
	FSP (3.2%)	2141 ^a^	3052	1.43	100	358 ^a^
High	Control	1740 ^c^	2832	1.63	100	255 ^c^
	FSP (1.6%)	1751 ^bc^	2854	1.63	100	256 ^bc^
	FSP (3.2%)	1821 ^b^	2992	1.64	97.2	264 ^b^
SEM		31.2	29.9	0.017	0	11.6
ANOVA						
Temp * FSP		0.050	0.072	0.170	0.863	0.043
Temp		0.001	0.001	0.001	0.932	0.001
FSP		0.012	0.885	0.415	0.912	0.013

^a–c^ Means with varying superscripts differ significantly (*p* ˂ 0.05). BWG, body weight gain; FI, feed intake; FCR, feed conversion ratio. ^a–c^ Means with varying superscripts differ significantly (*p* ˂ 0.05) BWG, body weight gain; FI, feed intake; FCR, feed conversion ratio.

**Table 3:** Carcass characteristics affected by supplementation with fennel seed powder under thermoneutral or heat stress conditions.

Temp.	Diet	Dressing (%)	Fat (%)	Leg (%)	Breast (%)	Intestine (%)	Gizzard (%)	Heart (%)	Liver (%)
Normal	Control	69.7	2.49	30.9	36.6	5.92 ^b^	3.32	0.92	2.72
	FSP (1.6%)	69.0	2.09	28.7	39.4	8.17 ^a^	3.34	0.84	2.91
	FSP (3.2%)	68.2	2.22	29.1	39.2	9.14 ^a^	3.43	0.95	2.99
High	Control	68.8	1.99	29.7	36.6	8.08 ^ab^	3.75	0.83	2.93
	FSP (1.6%)	68.7	1.98	29.8	38.5	7.70 ^ab^	3.56	0.65	2.75
	FSP (3.2%)	69.2	2.27	28.5	38.0	7.67 ^ab^	3.50	0.78	2.87
SEM		0.29	0.081	0.03	0.39	0.241	0.082	0.021	0.072
ANOVA									
Temp* FSP		0.385	0.361	0.173	0.791	0.003	0.652	0.640	0.510
Temp		0.894	0.248	0.658	0.355	0.856	0.136	0.001	0.867
FSP		0.713	0.489	0.069	0.030	0.03	0.902	0.021	0.801

^a,b^ Means with varying superscripts differ significantly (*p* ˂ 0.05).

**Table 4:** Broiler meat quality affected by supplementation with fennel seed powder under thermoneutral or heat stress conditions.

Temp	Diet	Nitrate Intake, mg/Chick	a*	b*	L*	Temp	pH
Normal	Control	62.8 ^e^	4.04 ^ab^	4.51	45.4	29.5 ^a^	6.69
	FSP (1.6%)	171.4 ^c^	5.31 ^a^	4.31	47.2	28.2 ^b^	6.74
	FSP (3.2%)	276.1 ^a^	3.47 ^bc^	4.88	45.6	27.9 ^b^	6.71
High	Control	56.2 ^f^	3.28 ^bc^	3.49	47.1	29.7 ^a^	6.73
	FSP (1.6%)	156.3 ^d^	2.56 ^bc^	2.89	47.9	29.3 ^b^	6.61
	FSP (3.2%)	265.7 ^b^	2.23 ^c^	2.59	48.0	29.1 ^b^	6.65
SEM		1.61	0.213	0.172	0.37	0.16	0.021
ANOVA							
Temp * FSP		0.115	0.029	0.098	0.612	0.007	0.509
Temp		0.005	0.0001	<0.0001	0.030	0.009	0.618
FSP		0.0001	0.016	0.596	0.311	0.139	0.932

^a–f^ Means with varying superscripts differ significantly (*p* ˂ 0.05).

**Table 5:** Economic efficiency of boilers affected by supplementation with fennel seed powder under thermoneutral or heat stress conditions.

Temp	Diet	Feeding Cost ($)	Total Cost ($)	Total Revenue ($)	Net Revenue ($)	Economic Efficiency (%)
Normal	Control	1.12 ^a^	1.92 ^a^	3.06 ^a^	1.14 ^a^	59.6
	FSP (1.6%)	1.10 ^a^	1.90 ^a^	2.98 ^b^	1.081 ^b^	57.0
	FSP (3.2%)	1.09 ^a^	1.89 ^a^	3.05 ^a^	1.17 ^a^	61.8
High	Control	1.00 ^c^	1.79 ^c^	2.48 ^e^	0.681 ^d^	37.9
	FSP (1.6%)	1.00 ^c^	1.79 ^c^	2.50 ^d^	0.696 ^d^	38.6
	FSP (3.2%)	1.05 ^b^	1.85 ^b^	2.60 ^c^	0.749 ^c^	40.6
SEM		0.032	0.030	0.056	0.0431	2.11
ANOVA						
Temp*FSP		0.022	0.022	0.001	0.023	0.134
Temp		0.001	0.001	0.001	0.001	0.001
FSP		0.270	0.270	0.001	0.002	0.013

^a–e^ Means with varying superscripts differ significantly (*p* ˂ 0.05). BWG, body weight gain; FI, feed intake; FCR, feed conversion ratio.
